# Overview on population screening for carriers with germline BRCA mutation in China

**DOI:** 10.3389/fonc.2022.1002360

**Published:** 2022-11-09

**Authors:** Huijun Lei, Min Zhang, Luyao Zhang, Kari Hemminki, Xiao-jia Wang, Tianhui Chen

**Affiliations:** ^1^ Department of Cancer Prevention/Zhejiang Cancer Institute, Cancer Hospital of the University of Chinese Academy of Sciences (Zhejiang Cancer Hospital), Hangzhou, China; ^2^ Institute of Basic Medicine and Cancer (IBMC), Chinese Academy of Sciences, Hangzhou, China; ^3^ School of Public Health, Hangzhou Medical College, Hangzhou, Zhejiang, China; ^4^ Department of Cancer Epidemiology and Prevention, Henan Engineering Research Center of Cancer Prevention and Control, Henan International Joint Laboratory of Cancer Prevention, The Affiliated Cancer Hospital of Zhengzhou University, Henan Cancer Hospital, Zhengzhou, China; ^5^ Biomedical Center, Faculty of Medicine, Charles University in Pilsen, Pilsen, Czechia; ^6^ Division of Cancer Epidemiology, German Cancer Research Center Deutsches Krebsforschungszentrum (DKFZ), Im Neuenheimer Feld, Heidelberg, Germany; ^7^ Department of Breast Medical Oncology, Cancer Hospital of the University of Chinese Academy of Sciences (Zhejiang Cancer Hospital), Hangzhou, China; ^8^ Department of Preventive Medicine, School of Medicine, Ningbo University, Ningbo, China

**Keywords:** population screening, BRCA, germline mutation, China, familial risk

## Abstract

Carriers with *BRCA1/2* germline pathogenic variants are associated with a high risk of breast and ovarian cancers (also pancreatic and prostate cancers). While the spectrum on germline *BRCA* mutations among the Chinese population shows ethnic specificity, the identification of carriers with germline *BRCA* mutation before cancer onset is the most effective approach to protect them. This review focused on the current status of *BRCA1/2* screening, the surveillance and prevention measures, and discussed the issues and potential impact of *BRCA1/2* population screening in China. We conducted literature research on databases PubMed and Google Scholar, as well as Chinese databases CNKI and Wangfang Med Online database (up to 31 March 2022). Latest publications on germline *BRCA1/2* prevalence, spectrum, genetic screening as well as carrier counseling, surveillance and prevention were captured where available. While overall 15,256 records were retrieved, 72 publications using germline *BRCA1/2* testing were finally retained for further analyses. Germline *BRCA1/2* mutations are common in Chinese patients with hereditary breast, ovarian, prostate and pancreatic cancers. Within previous studies, a unique *BRCA* mutation spectrum in China was revealed. Next-generation sequencing panel was considered as the most common method for *BRCA1/2* screening. Regular surveillance and preventive surgeries were tailored to carriers with mutated-*BRCA1/2*. We recommend that all Chinese diagnosed with breast, ovarian, pancreatic or prostate cancers and also healthy family members, shall undergo *BRCA1/2* gene test to provide risk assessment. Subsequently, timely preventive measures for mutation carriers are recommended after authentic genetic counseling.

## Introduction

Breast cancer genes *BRCA1* and *BRCA2* are tumor suppressor genes that function in DNA double-strand break repair in the homologous recombination pathway. Mutated *BRCA1/2* genes can cause *BRCA1/2* protein deficiency and genome instability (1). Since the identification of *BRCA1* and *BRCA2* genes in the 1990s as the landmarks of hereditary breast and ovarian cancer, human beings enter the era of cancer genetic testing. Female *BRCA* mutation carriers have 60-80% of lifetime risk of developing breast cancer and 20-40% of risk of ovarian cancer (2). Mutation in *BRCA* is also associated with an increased risk of prostate and pancreatic cancers (3). In addition, *BRCA* pathogenic mutation carriers are significantly associated with increased disease risk for three additional cancers, including biliary tract cancer, gastric cancer, and esophageal cancer (4). Notably, *BRCA1* pathogenic variants carriers have a 4.30, 2.36 and 2.17-fold elevated lifetime risk of the male breast, pancreatic and stomach cancers compared to non-carriers. *BRCA2* pathogenic variants carriers have 44.0, 3.69, 3.34 and 2.22-fold elevated lifetime risk of the male breast, stomach, pancreatic and prostate cancers compared to non-carriers, respectively (5).

**Figure 1:**
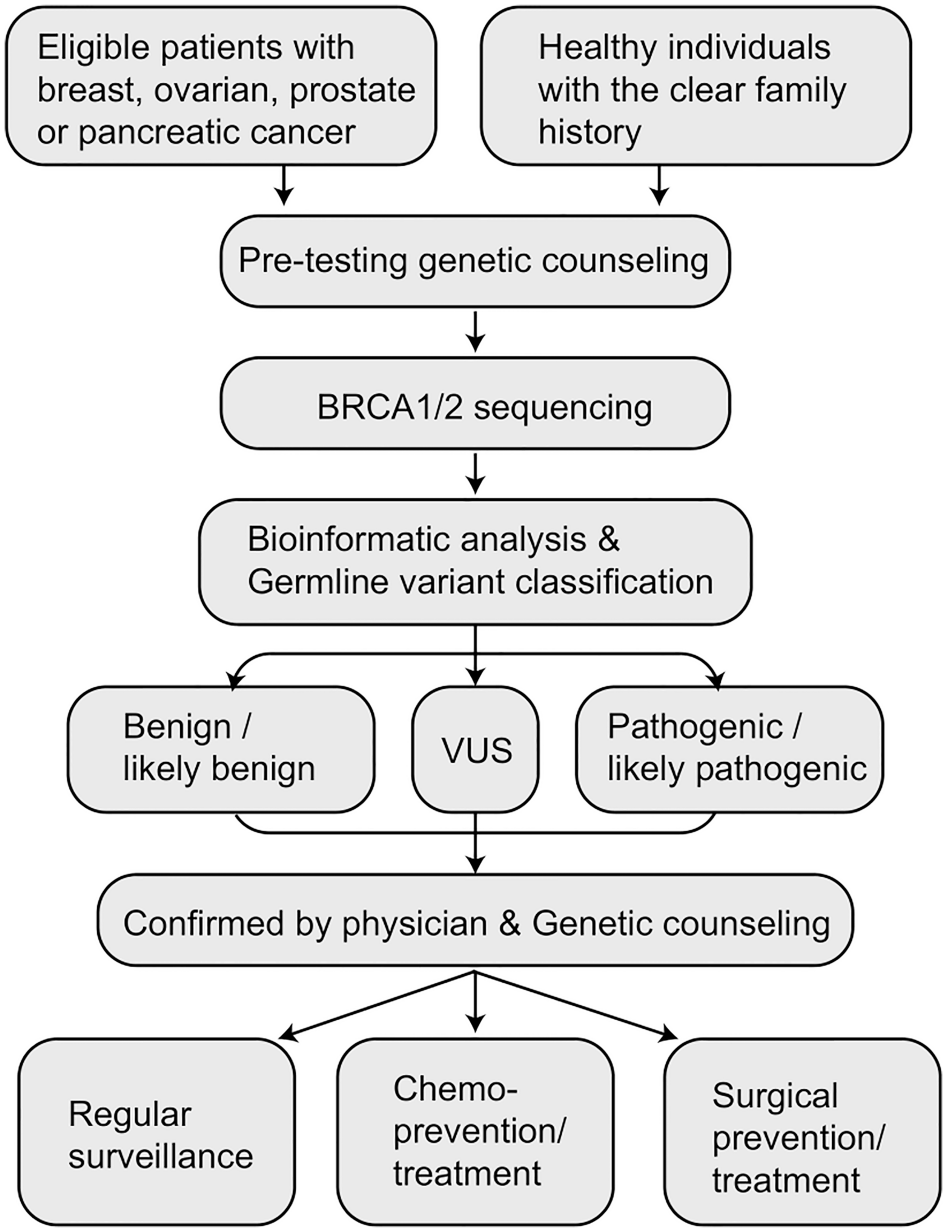
The procedure of population screening for BRCA germline mutation carriers in China (7–11).

Early detection and prevention have been proven to reduce cancer incidence and mortality (while increasing cancer survival) in mutation carriers (3, 6). Therefore, identifying *BRCA* mutation carriers is important to reduce cancer risk. In this review, we conducted literature research on PubMed, Google Scholar and Chinese databases about germline *BRCA1/2* mutation in the Chinese populations included literature published up to 31 March 2022. A total of 15,256 publications were obtained: PubMed (n=856), Google Scholar (n=6,153), CNKI (n=4,935) and Wangfang Med Online database (n=3,312). After removing duplicates, selecting the title and the abstract and carefully reading the whole paper, 72 publications related to germline *BRCA1/2* testing were finally included. Based on the comprehensive literature review, we discuss population screening approaches for comprehensive identification of the *BRCA* mutation carriers in the Chinese population and propose the ideal procedure for achieving the goals in China (**Figure 1**) (7–11).

## Overall prevalence and spectrum of *BRCA* mutation carriers in China and elsewhere

In the general Chinese population, the prevalence of pathogenic *BRCA1/2* variation has been reported to range from 0.29 to 1.10% (0.02 to 0.34% for *BRCA1* and 0.11 to 0.27% for *BRCA2*) (12–15).

The prevalence of *BRCA1/2* in the general population varies by country and ethnicity (16, 17). It was 0.18% in a Malaysian group of 2,809 individuals, 0.26% among 22,731 Japanese, 0.38% in a Mexican population of 3,985 individuals, 0.53% in 50,726 US people and 2.17% in the Ashkenazi Jewish population, which is the highest (18–22). The prevalence of *BRCA1/2* mutation in the general Chinese population is intermediate.

The spectrum of *BRCA* variation in Chinese is rather different from those in non-Chinese populations (15, 17). It was reported that approximately 38-41.4% of *BRCA* variants were only present in the Chinese population (23, 24). Even when compared to neighboring India, only 4.1% and 0.4% of shared *BRCA1* and *BRCA2* variants were found in both populations (24).

In a large-scale cohort with 1,245 pathogenic variants identified, 48 most common pathogenic *BRCA1/2* variants (39.86% of total) were not reported as common variants in Caucasians (15). The pathogenic variant *BRCA1* c.5470_5477del was determined as a founder mutation in the Chinese Han population (25, 26). Interestingly, another systematic review with 2,128 *BRCA1/2* variants derived from 35,178 Chinese individuals from 23 provinces also reported that c.5470_5477del ranked as the highest frequency of all *BRCA1* variants identified while the c.3109C>T ranks highest in *BRCA2* (12). Further, *BRCA1* c.3770_3771delAG was the most common variant in Chinese ovarian cancer patients (27). The proportions of frameshift, nonsense, splice and missense mutations in Chinese ovarian cancer patients were determined as 51.2%, 39.3%, 7.1% and 2.4%, respectively (28). But the founder mutations in other ethnic populations, such as *BRCA1* c.66_67delAG, *BRCA2* c.5946delT in Ashkenazi Jewish, *BRCA* 1 c.303T>G, c.1623dupG in African, *BRCA1* c.390C>A in Japanese and Korean and many other founder mutations in different non-Chinese populations, were absent or at low prevalence in Chinese population (24).

## Prevalence of *BRCA* mutations in different populations in China

We summarized the prevalence of *BRCA1/2* germline mutation in different populations from large-scale cohort studies published within five years (**Table 1**). A total of 41 studies were included for further analysis (13–15, 27, 29–35, 64).

**Table 1:** Summary of BRCA prevalence studies from China in recent 5 years.

Reference*	Year	BRCA1/2(+) number	BRCA1/2 (+) rate (%)	BRCA1 (+) N	BRCA1 (+) rate (%)	BRCA2 (+) N	BRCA2 (+) rate (%)	Sequencing methods	Singlecenter or multicenter	Regions	Study Population	Select criteria	Size	Age range	Median age	Average age
(13)	2021	43	0.38	13	0.11	30	0.26	NGS panel**+sanger	multicenter	nation-wide	general	healthy population	11386	>19	–	F34.8/M43.0
(15)	2021	71	1.10	–	–	–	–	NGS panel+sanger	multicenter	–	general	healthy population	6434	–	–	34.8
(14)	2021	18	0.29	1	0.02	17	0.27	NGS panel+sanger	singlecenter	Macau	general	healthy population	6314	–	–	F42.0/M41.0
(29)	2017	4	0.38	–	–	–	–	NGS panel+sanger	multicenter	Shanghai, Fujian	general	healthy population	1043	–	–	–
(27)	2018	8	0.45	6	0.34	2	0.11	NGS panel+sanger	multicenter	–	general	healthy population	1763	–	–	37.5
(30)	2019	138	15.65	89	10.09	49	5.56	NGS multi-gene panel(21***)+sanger	singlecenter	Guangdong	high risk population	high risk of HBOC	882	13-80	–	47.0
(31)	2021	23	19.83	11	9.48	12	10.34	NGS multi-gene panel (43)+sanger	singlecenter	Tianjin	breast cancer/high risk population	familial patients and their direct relatives	116	26-76	51	50.0
(32)	2017	31	23.31	18	13.53	13	9.77	NGS panel+duplicate independent PCR	singlecenter	Zhejiang	breast/ovarian cancer	familial	133	22-74	–	43.0
(15)	2021	1174	5.53	–	2.3	–	3.1	NGS panel+sanger	multicenter	–	breast cancer	unselected	21216	–	–	49.7
(33)	2017	428	5.29	146	1.81	285	3.53	NGS multi-gene panel (62)+sanger	singlecenter	Beijing	breast cancer	unselected	8085	–	–	–
(34)	2019	148	5.34	74	2.67	76	2.74	NGS panel+sanger	singlecenter	Zhejiang	breast cancer	unselected	2769	–	–	49.4
(35)	2020	29	5.53	11	2.10	18	3.44	NGS multi-gene panel (62)	singlecenter	Guangdong	breast cancer	unselected	524	22-86	–	49.2
(36)	2021	13	3.82	5	1.47	8	2.35	NGS panel	singlecenter	Guangdong	breast cancer	unselected	340	–	–	49.9
(37)	2017	17	5.43	5	1.60	12	3.83	NGS panel+sanger	singlecenter	Hunan	breast cancer	unselected	313	21-84		51.2
(29)	2017	232	9.06	105	4.10	128	5.00	NGS panel+sanger	multicenter	Shanghai, Fujian	breast cancer	familial	2560	–	–	–
(33)	2017	146	18.14	59	7.33	87	10.81	NGS multi-gene panel (62)+sanger	singlecenter	Beijing	breast cancer	familial	805	–	–	–
(38)	2019	94	19.54	70	14.55	24	4.99	NGS multi-gene panel (22)+sanger	multicenter	nation-wide (28 centers)	breast cancer	familial	481	19-77	47	–
(33)	2017	198	3.32	56	0.94	142	2.38	NGS multi-gene panel (62)+sanger	singlecenter	Beijing	breast cancer	sporadic	5963	–	–	–
(39)	2019	159	16.97	82	8.75	81	8.64	NGS panel (40)	multicenter	nation-wide (26 centers)	breast cancer	high risk	937	8-77	–	37.5
(40)	2018	40	8.33	6	1.25	34	7.08	NGS multi-gene panel (20)+sanger	singlecenter	Guangdong	breast cancer	high risk	480	17-82	–	41.8
(41)	2017	35	7.94	9	2.04	26	5.90	SNaPshot/NGS/MLPA+Sanger	multicenter	Hongkong	breast cancer	high risk	441	18-87	–	47.1
(42)	2018	76	17.39	–	–	–	–	NGS panel	multicenter	nation-wide (18 centers)	breast cancer	high risk	437	–	–	–
(29)	2017	15	3.48	–	–	–	–	NGS panel+sanger	multicenter	Shanghai, Fujian	breast cancer	high risk	431	–	–	–
(39)	2019	18	8.33	11	5.09	7	3.24	NGS panel+sanger	multicenter	Inner Mongolia, Jilin	breast cancer	high risk	216	21-67	42	–
(43)	2021	67	18.87	–	–	–	–	NGS panel+sanger	singlecenter	Shanghai	breast cancer	early-onset TNBC	355	24-40	34	–
(44)	2020	85	6.31	24	1.78	61	4.53	–	singlecenter	Fujian	breast cancer	early-onset	1347	<40	–	–
(45)	2019	4	14.81	2	7.41	2	7.41	NGS panel+sanger	singlecenter	Sichuan	breast cancer	early-onset	27	23-40	–	32.0
(46)	2020	35	10.77	24	7.38	9	2.77	–	singlecenter	Shanghai	breast cancer	TNBC	325	–	–	–
(47)	2021	26	20.97	20	16.13	6	4.84	NGS panel+MLPA	singlecenter	Shanghai	breast cancer	TNBC	124	24-55	46	–
(45)	2019	1	3.70	0	0.00	1	3.70	NGS panel+sanger	singlecenter	Sichuan	breast cancer	non-early-onset	27	41-68	–	52.0
(48)	2018	48	8.07	17	2.86	31	5.21	NGS panel+sanger	multicenter	Guangdong, Shandong, Chongqing	breast cancer	–	595	22-80	–	48.0
(28)	2019	129	23.58	84	15.36	45	8.23	NGS panel+sanger	multicenter	Shandong	ovarian/fallopian tube/peritoneal cancer	unselected	547	–	–	–
(49)	2021	14	22.58	12	19.35	2	3.23	NGS multi-gene panel+sanger/qPCR	singlecenter	Beijing	ovarian/fallopian tube/peritoneal cancer	unselected	62	34-82	56	–
(27)	2019	297	26.26	227	20.07	70	6.19	NGS panel+sanger	multicenter	nation-wide	ovarian cancer	unselected	1131	9-24	–	51.5
(50)	2017	235	28.45	172	20.82	63	7.63	NGS panel+sanger/qPCR	multicenter	Shanghai, Beijing, Shandong, Guangdong, Sichuan	ovarian cancer	unselected	826	–	52	–
(51)	2017	41	23.84	35	20.35	28	16.28	NGS panel	singlecenter	Beijing	ovarian cancer	unselected	172	18-81	–	52.5
(41)	2020	13	8.39	9	5.81	4	2.58	SNaPshot/NGS panel/MLPA+Sanger	multicenter	Hong Kong	ovarian cancer	unselected	155	9-85	**-**	44.7
(52)	2022	64	32.82	37	18.97	32	16.41	NGS panel	singlecenter	Guangdong	ovarian cancer	Hakka people	195	–	–	–
(53)	2017	30	26.09	24	20.87	6	5.22	NGS panel+MLPA	singlecenter	Shanghai	ovarian cancer	high grade serous ovarian cancer	115	38-79	51	–
(54)	2018	153	16.70	120	13.10	36	3.93	NGS panel	multicenter	nation-wide (25 centers)	ovarian cancer	epithelial ovarian cancer	916	20-81	–	54.2
(55)	2021	36	30.51	31	26.27	5	4.24	NGS multi-gene panel (18) +sanger/qPCR	singlecenter	Anhui	ovarian cancer	epithelial ovarian cancer	118	31-79	–	52.0
(56)	2017	9	18.00	3	6.00	6	12.00	WES+sanger	singlecenter	Beijing	ovarian cancer	epithelial ovarian cancer	50	25-79	53	–
(57)	2022	–	–	–	0.5	–	1.9	NGS multi-gene panel(381/733)	singlecenter	Shanghai	pancreatic cancer	unselected	1080	20-87	60	–
(58)	2021	–	–	–	0	–	0.33	NGS multi-gene panel(566/764)	singlecenter	Shanghai	pancreatic cancer	unselected	608	–	–	–
(59)	2021	10	5.13	1	0.51	9	4.62	NGS multi-gene panel(150/381/437)	singlecenter	Sichuan	pancreatic cancer	pancreatic ductal adenocarcinoma	195	27-79	59	–
(60)	2021	86	4.68	7	0.38	79	4.30	WES/NGS multi-gene panel(2~618)	multicenter	Shanghai, Hong Kong, Sichuan, Guangdong	prostate cancer	unselected	1836	61-73	–	67.0
(61)	2019	22	7.01	2	0.64	20	6.37	WES/NGS multi-gene panel(63/499/618)	singlecenter	Shanghai	prostate cancer	–	314	34-84	64	63.4
(62)	2021	–	–	–	0.4	–	5.3	WES	singlecenter	Shanghai	prostate cancer	unselected	246	57-69	65	–
(63)	2021	10	0.46	1	0.05	9	0.42	NGS panel(365genes+25 genes frequently re-arranged)	–	–	colorectal cancer	unselected	2160	–	–	–
(64)	2019	17	4.76	–	–	–	–	NGS panel(450genes+36 genes frequently re-arranged)	singlecenter	Beijing	liver cancer	unselected	357	16-88	–	56.0

*Only large-scale studies (n>50 in unselected cancer or n>300 in general population) are included.

**NGS panel mainly refers to the 2 gene panel (BRCA1 and BRCA2).

***The number of cancer susceptibility genes contained in NGS multi-gene panel.

In the Chinese cancer patients, a study showed that the prevalence rate was 5.53% for *BRCA1/2* (43.7% in *BRCA1* and 56.3% in *BRCA2*) in unselected breast cancer patients (15). In comparison, a higher prevalence of 9.06-19.54% for *BRCA1/2* mutation was observed in familial breast cancer patients (29, 31, 33). 60% of breast cancer patients carrying *BRCA1* deleterious mutation were classified as triple-negative breast cancer, while only 10 to 20% were triple-negative breast cancer in unselected cancer patients (33, 34). Patients with *BRCA1/2* mutated breast cancer generally show an earlier age of onset, on average 5 to 8 years earlier than patients with sporadic breast cancer (33, 35). *BRCA1/2* pathogenic variants are also enriched in bilateral breast cancer and patients with family history of breast or other cancers (33, 34). In unselected ovarian cancer patients, *BRCA1* pathogenic variants were more common compared to *BRCA2* (20.07% *vs*. 6.19%) (27). Pathogenic mutations in *BRCA1* genes were more related to a younger diagnosis age, serous ovarian carcinoma and hereditary breast and ovarian cancer syndrome (HBOC) (27). Among prostate cancer patients carrying germline mutations, *BRCA2* is the most common mutated gene among DNA damage repair pathway genes. The prevalence of *BRCA1* and *BRCA2* pathogenic variants was 0.38% and 4.30%, respectively, in prostate cancer patients (55). *BRCA2* was also reported as the most frequent gene in the germline in pancreatic cancer patients, with a prevalence rate of 1.9%; the frequency of *BRCA1* variants was 0.5% (57). However, there is a lack of multicenter studies on *BRCA* mutations in pancreatic cancer. It is worth noting that the actual prevalence may be higher than what is now predicted because the data for pathogenic variants interpretation are mainly from non-Chinese populations. In addition, most of the studies summarized in the **Table 1** examined only single nucleotide variants and indels and did not detect mutations of large genomic rearrangements (LGRs). It is possible that many unknown pathogenic variants have not been identified.

Despite increasing data from large-scale and multicenter *BRCA* studies having been reported, no *BRCA* data is reported for the Chinese living in many remote areas (12). Most *BRCA1/2* prevalence studies were from cities with relatively developed economies and medical care, such as Beijing, Shanghai, Hong Kong, Guangdong, Zhejiang and Sichuan. Possibly because genetic testing is not yet covered by basic medical insurance, patients in economically developed regions are more likely to afford expensive genetic testing. Meanwhile, economically and medically developed regions have more medical resources, such as genetic testing facilities and genetic counseling services (65). The bias is also because these regions have more investigators and research funds and are more likely to conduct clinical studies. However, considering the regional and ethnic specificity of *BRCA* gene variation, substantial efforts are needed to generate a comprehensive *BRCA* variation map for the Chinese population.

## Methodologies for population *BRCA* screening in Chinese population

In the mid-1990s, the identification of the relationship between *BRCA1*/*2* mutation and cancer risk heralded the era of genetic testing for susceptibility to cancer. Subsequently, germline *BRCA1* and *BRCA2* mutations were extensively studied in the Caucasian populations, and associations with breast and ovarian cancers were established (66). Sanger sequencing has been widely used in *BRCA* variant identification since the 1990s, but the development of next-generation sequencing (NGS) revolutionized the detection strategy due to its affordability and efficiency.

NGS, including whole-genome sequencing (WGS), whole-exome sequencing (WES) and panel sequencing, have facilitated *BRCA* mutation research (67). Also because of the policy support in 2015, large-scale *BRCA* studies in China have increased rapidly since then (12, 42). Due to the lack of hotspot variation, NGS is currently the optimal option for *BRCA1/2* genetic testing in the Chinese population. NGS panel test is widely implemented for clinical *BRCA* test in China in recent years. The two-gene panel is a more preferred option for the general population, breast cancer and ovarian cancer patients, while pancreatic, prostate and other cancer patients tend to be suggested with the multi-gene panel in China (**Tables 1**, **2**).

**Table 2:** Summary of guidelines and consensus about BRCA1/2 genetic testing in recent 5 years in China.

Reference	Title	Organization	Year	Language	Target population	Recommended population for genetic testing
(10)	Guidelines of Chinese Society of Clinical Oncology (CSCO) - Pancreatic Cancer (2022 Edition)	Chinese Society of Clinical Oncology Guidelines Working Committee	2022	Chinese	pancreatic cancer patient	Germline genetic testing is recommended for all patients diagnosed with pancreatic cancer
(68)	Clinical Practice Guideline of BRCA1/2 Testing for Patients with Breast Cancer: Chinese Society of Breast Surgery (CSBrS) Practice Guideline 2021	Chinese Society of Breast Surgery (CSBrS)	2021	English	breast cancer patient	1.Breast cancer diagnosed ≤45 years old; 2.Breast cancer diagnosed 46 to 50 years old with one or more of the following: An additional breast cancer primary at any age; ≥1 close blood relative† with breast cancer at any age; An unknown or limited family history; 3.Diagnosed ≤60 years old with triple negative breast cancer; 4.Breast cancer diagnosed at any age with one or more of the following: ≥1 close blood relative† with breast cancer diagnosed 50 years old; ≥1 close blood relative† with ovarian carcinoma/metastatic prostate cancer/pancreatic cancer/male breast cancer; ≥2 additional diagnoses of breast cancer at any age in patient and/or in close blood relatives; Personal history of ovarian carcinoma/pancreatic cancer; 5.Male breast cancer; 6.Patients with HER2negative recurrent metastatic breast cancer; 7.BRCA1/2 pathogenic/likely pathogenic variant were detected in tumor tissues; 8.Individual from a family with a known BRCA1/2 pathogenic/likely pathogenic variant; 9.Ovarian carcinoma; 10.High-grade prostate cancer with one or more of the following: ≥1 close blood relatives† with ovarian carcinoma/pancreatic cancer/metastatic prostate cancer/breast cancer <50 years old; ≥2 close blood relatives† with breast/prostate cancer (any grade) at any age.
(9)	Expert Consensus on Clinical Treatment of Familial Hereditary Tumors in China (2021 Edition)-Familial Hereditary Breast Cancer	China Anti-Cancer Association, Familial Hereditary Cancer Committee	2021	Chinese	breast cancer patient	a. Individuals with a history of breast cancer with any of the following conditions: 1. Age at presentation ≤ 50 years. 2. Triple-negative breast cancer. 3. Male breast cancer. 4. Age at presentation >50 years and ≥1 other breast, ovarian, pancreatic or prostate cancer in the family. 5. Patients with operable primary HER-2 negative breast cancer with high risk of recurrence, regardless of family history of breast cancer or other tumors. 6. HER-2 negative metastatic breast cancer. b. Individuals with a history of breast cancer, regardless of whether they have any of the following conditions: 1. Immediate family members with known pathogenic or potentially pathogenic mutations in the BRCA1/2 gene. 2. A male breast cancer patient in the family. 3. Healthy individuals* may be tested if they have ≥2 cases of breast cancer in the family; or ≥2 tumor types including breast, ovarian, pancreatic, or prostate cancer with at least 1 breast cancer in the family.(*However, it is still recommended that individuals with cancer in the family be tested as a priority, especially those with early age of onset and multiple primary tumors; healthy individuals in the family should be considered for testing only when patients are not available.)
(69)	Consensus of Chinese Experts on Hot Isssues in GneticTesting of Advanced Breast Cancer (2021 edition)	International Medical society, Chinese Anti-cancer Association	2021	Chinese	advanced breast cancer patient	Patients with advanced breast cancer who are financially eligible and have accessible pathological specimens.
(8)	Expert Consensus on Clinical Treatment of Familial Hereditary Tumors in China (2021 Edition)-Familial Hereditary Prostate Cancer	China Anti-Cancer Association, Familial Hereditary Cancer Committee	2021	Chinese	prostate cancer patient	Germline mutation testing for DNA damage repair genes, including BRCA2, BRCA1, ATM, PALB2, CHEK2, MLH1, MSH2, MSH6, and PMS2, is recommended for people at genetic risk for prostate cancer who meet any of the following criteria: 1.Known family members carry pathogenic mutations in the above genes. 2. Patients with a clear family history of tumors and multiple cases in the same family including bile duct cancer, breast cancer, pancreatic cancer, prostate cancer, ovarian cancer, colorectal cancer, endometrial cancer, gastric cancer, renal cancer, melanoma, small intestine cancer and uroepithelial cancer, especially if their age of diagnosis is ≤ 50 years; and patients with a brother, father or other family members diagnosed with prostate cancer or died of prostate cancer before the age of 60 years. 3.With a suspicious or unknown family history, recommended after adequate genetic counseling evaluation. 4. Tumor tissue testing reveals no germline verification of the above gene pathogenic mutation. 5.Intraductal carcinoma and ductal adenocarcinoma. 6.High risk and above, locally progressive and metastatic prostate cancer.
(7)	Expert Consensus on Clinical Treatment of Familial Hereditary Tumors in China (2021 Edition)-Familial Hereditary Ovarian Cancer	China Anti-Cancer Association, Familial Hereditary Cancer Committee	2021	Chinese	ovarian cancer patient	1.Patients with primary epithelial ovarian cancer; 2.Patients with recurrent epithelial ovarian cancer; 3.Individuals with germline mutations detected in ovarian cancer, further “ cascade testing” of their family line is required
(70)	Chinese Expert Consensus on Genomic Testing of Prostate Cancer Patients (the 2020 edition)	China Anti-Cancer Association Genitourinary Cancer Committee	2020	Chinese	prostate cancer patient	a. To provide genetic counseling for the purpose of 1. Patients with a clear family history of prostate cancer who have not undergone risk assessment at first diagnosis or who are at very low to intermediate risk; patients with unknown or unclear family history need to be guided by oncologic genetic counseling to consider the need for testing 2. Patients with high-risk or very high-risk prostate cancer 3. Patients with locally progressive (N1) or metastatic (M1) prostate cancer, intraductal carcinoma of the prostate (IDC-P) or ductal adenocarcinoma of the prostate (DAP) pathology Prostate cancer patients 4. Patients with prostate cancer whose tumor tissue testing has identified mutations associated with risk of tumor development and who lack verification of germline variants will be considered for testing after genetic counseling recommendations. b. For the purpose of making treatment decisions 1. Patients with metastatic castration-resistant prostate cancer (mCRPC)
(11)	Guideline on Next⁃Generation Sequencing⁃Based BRCA1/2 Testing (2019)	Working Group of Guideline on Next⁃Generation Sequencing⁃Based BRCA1/2 Testing (2019)	2019	Chinese	not specific	a.To assess genetic risk, genetic counseling and germline BRCA1/2 gene testing are recommended for relevant high-risk populations, including (1) individuals from families with pathogenic/probably pathogenic mutations in the BRCA1/2 gene; (2) patients with pathogenic/probably pathogenic mutations in the BRCA1/2 gene identified by tumor testing but for whom it is not clear whether they are germline mutations; (3) all newly diagnosed patients with ovarian cancer, fallopian tube cancer and primary peritoneal cancer; (4) breast cancer patients with age of onset of 40 years or younger, triple negative breast cancer patients with age of onset of 60 years or younger, all male breast cancer patients; (5) all newly diagnosed pancreatic cancer patients; (6) patients with high risk and above, N1 and M1 prostate cancer, prostate intraductal cancer patients; (7) breast cancer and prostate cancer patients; (8) individuals with one or more 1st or 2nd degree blood relatives meeting the above testing criteria, etc. b.To guide the selection of subsequent treatment options, (1) germline and/or somatic BRCA1/2 gene testing is recommended for all newly diagnosed ovarian, fallopian tube, and primary peritoneal cancer patients, and BRCA1/2 gene testing using newly obtained tumor tissue is considered after recurrence); (2) germline BRCA1/2 gene testing is recommended for HER2-negative advanced breast cancer patients when considering chemotherapy gene testing; (3) germline and/or somatic cell BRCA1/2 gene testing is recommended for patients with locally advanced and metastatic pancreatic cancer at the time of diagnosis); (4) testing for germline and somatic cell variants containing at least DNA damage response genes such as BRCA1/2 is recommended for all patients with metastatic desmoplastic resistant prostate cancer
(71)	Expert Consensus on BRCA1/2 Gene Testing and Clinical Application in Chinese Breast Cancer Patients (2018 edition)	Chinese Medical Doctor Association, Chinese Society of Precision Medicine, Breast Cancer Committee	2018	Chinese	breast cancer patient	Breast cancer patients: ≤40 years of age onset ≤50 years of age with: (1) second primary breast cancer (2) ≥1 of the following family history criteria: ① ≥1 consanguineous relative with a history of breast cancer at any age; ② ≥1 consanguineous relative with a history of pancreatic cancer; ③ ≥1 relative with a history of prostate cancer (Gleason score ≥7); ④ Unknown or limited family history ≤60 years of age with (2) ≥1 consanguineous relative with a history of breast cancer at ≤50 years of age; (3) ≥1 consanguineous relative with a history of ovarian cancer; (4) ≥3rd degree relative with breast and/or ovarian cancer and ≥2 consanguineous relatives with breast cancer (at least 1 of whom is ≤50 years of age) and/or ① Family history of male breast cancer in a consanguineous relative; ② ≥2 consanguineous relatives with pancreatic and/or prostate cancer of any age (Gleason score ≥7); ③ Known familial pathogenic BRCA1/2 gene mutation
(72)	Guidelines for the Diagnosis and Treatment of Ovarian Malignancies (4th edition)	China Anti-Cancer Association Gynecology Cancer Committee	2018	Chinese	ovarian cancer patient	Genetic testing is recommended for individuals with one or more of the following: (1) Known BRCA1/2 mutation in the family. (2) Personal history of ovarian cancer or other HBOC-related tumors with age at diagnosis ≤50 years. (3) Have HBOC-associated tumor with age at diagnosis ≤ 60 years and a second primary tumor, or triple-negative breast cancer, or ≥ 1 close relative with HBOC-associated tumor (4) ≥2 close relatives with HBOC-associated tumors. (5) Male breast cancer patients, or male close relatives with breast cancer; BRCA1/2 mutation detected in tumor tissue, but germline analysis not performed.

Because of its accuracy, Sanger sequencing remains to be a gold standard for detecting *BRCA* variants and validating NGS-detected *BRCA* variants and can be used in confirming the findings (67). Practical test- and laboratory-specific criteria have been proposed for confirmation strategy to facilitate timely delivery of clinical accuracy (73).

Many studies involving different populations have shown that LGRs in *BRCA1/2* can be identified in HBOC (74–76). Multiplex ligation-dependent probe amplification (MLPA) is a cheap, sensitive and reliable method for detecting gene rearrangements (77). In the eastern Chinese population, 2.9% of HBOC patients without detectable *BRCA1/2* small pathogenic variants were identified harboring LGRs in *BRCA* (78). The data are similar to those from the Myriad data set with high-risk patients, most of whom were diagnosed with early-onset ovary cancer or male breast cancer. The study reported an overall *BRCA1/2* mutation rate of 23.8%, of which 9.9% were LGRs. Thus, large genomic rearrangement testing is recommended if the NGS result is negative for high-risk populations to avoid the missed diagnosis of *BRCA1/2* mutation carriers (71).

## Problems related to panel sequencing

NGS panel, the recommended method for *BRCA1/2* testing in clinical practice, still has some problems. First, variants of uncertain significance (VUS) increase with testing a larger panel or increasing genome sequencing length, making *BRCA1* and *BRCA2* interpretation more complex (79). For example, 24.7% of variants reported in the general population and 43.8% reported in breast cancer were identified as VUS, respectively (13, 31). Classification of VUS as pathogenic or benign variants has important clinical implications for cancer diagnosis and treatment (80). The methods to identify VUS as pathogenic or benign need to become more efficient and accurate, considering the huge abundance of VUS. *BRCA1/2* variants interpretation mainly follows the Chinese expert consensus on *BRCA1/2* variant interpretation (2021 version) (81) and the American College of Medical Genetics and Genomics and the Association for Molecular Pathology (ACMG/AMP) guideline (82) in China.

Nevertheless, the population, disease-specific and sequence databases commonly used for interpretation contain few Chinese or Asian data. Lacking Chinese ethnic-specific data makes variant interpretation highly reliant on the peer-reviewed literature, which is also limited. This challenging context prevents many pathogenic variants from being identified and the VUS increases even more (83). Suggesting a new classification system for Chinese is needed, including but not limited to the databases based on Chinese populations and biological function identification of Chinese specific variants. Chinese Familial & Hereditary Cancer Susceptibility Gene Mutation Database (CFCSG-database) is one of the biggest cancer susceptibility gene mutation databases based on Chinese population. But the amount of *BRCA1* and *BRCA2* variants is still limited in it. More large-scale population studies and function studies of *BRCA1/2* mutation in Chinese are needed to obtain more evidence to optimize the mutation interpretation.

As new shreds of evidence accumulate, the variant classification could be change over time. A study of 21,216 Breast cancer patients and 6,434 healthy controls performed VUS reclassification in the cohort. After the reclassification, 7 VUS were re-grouped into benign, which reduced the VUS ratio in both patient and healthy control (from 9.8 to 7.9% and from 6.9 to 5.3%) (15), indicating that the evidence should frequently be updated for VUS reclassification, and emphasizing the VUS carriers should be followed up.

Another notable issue concerns the price of BRCA mutation testing (84). Currently the price of BRCA mutation testing for a single sample in China is roughly 300 dollars ($), which is only paid by the patient side and not covered by the government side through basic medical insurance (85). Actually, the price of a single BRCA mutation testing is too high for the majority of ordinary Chinese. Therefore, financial investment from the Chinese government side is necessary to promote the widespread of BRCA mutation testing across China, e. g., Chinese government could offer reimbursement through Chinese basic medical insurance system for the high-risk population who took BRCA mutation testing. Additionally, evidence shows population-based BRCA mutation screening is also cost-effective for Chinese data with an incremental cost-effectiveness ratio of $18,066 from a societal perspective and $23,485 from a payer perspective per quality-adjusted life year (86).

In fact, while these issues are prominent in China, they also exist in many other countries and need to be addressed through collective efforts.

## Genetic counseling for *BRCA* mutation carriers in China

Currently, the principles of *BRCA* mutation detection in China mainly refers to the guideline on next-generation sequencing-based *BRCA1/2* testing (2019) (11), the US National Comprehensive Cancer Network (NCCN) guidelines (87) and the European Society of Medical Oncology (ESMO) guidelines (88), as well as other Chinese expert consensus on specific cancers or genetic testing (summarized in **Table 2**). We summarized the criteria proposed in 10 different guidelines and consensuses for Chinese population *BRCA* screening in recent 5 years (7–11, 68–72).

Genetic counseling is essential in pre- and post-sequencing stage for the test individuals. The purpose is to accurately estimate the probability of cancer susceptibility gene mutations (89) and offer early prevention advice and medical management such as regular surveillance, chemoprevention or surgical prevention for *BRCA* mutation carriers (27, 28). In a study with 839 breast cancer patients and 510 relatives, who are considered high-risk populations, 86.4% and 63.8% cases showed a strong willingness to accept genetic counseling and genetic testing, respectively (90). For those high-risk populations who are willing to do the genetic testing of *BRCA1/2*, the mutation rate was 19.9%. Despite the high willingness, most of the high-risk individuals lacked knowledge of cancer inheritance (90). We are glad to find out that another study exhibited that 79% of germline mutation carriers were aware of the risk and the importance of surveillance, while 56% accepted preventive interferences after genetic counseling on gynecologic tumors (91).

However, the development of cancer genetic counseling in China is in its beginning. Unlike some developed countries where specialized and certified genetics health professionals are available (92), cancer genetic counseling relies heavily on clinicians. Setting up standardized workflows and training eligible counselors is pivotal for promoting genetic counseling in China. Although the “oncologist-led *BRCA* consultation” mode has improved access to cancer genetic testing in developing countries (93), specialized cancer genetic counselors are urgently needed. Organizations like the Chinese Board of Genetic Counseling and others are now dedicated to training genetic counselors in more than 15 provinces across China (65). Meanwhile, the Chinese Anti-Cancer Association is urging hospitals nationwide to set up cancer genetic counseling clinics to accommodate the increased demand for counseling. Still, the training projects and qualified counselors are minimal and lack statistics.

## Regular surveillance, prevention and treatment for *BRCA* mutation carriers in China

After genetic testing, the frequency of regular surveillance for female mutation carriers was significantly higher compared to non-carriers, according to the report on high-risk southern Chinese females (94).

Early-stage breast cancer lacks apparent signs and symptoms. Possible symptoms of breast cancer can be skin dimpling, red or thickening, nipple retraction and lymph nodes swelling. But a painless hard lump with irregular edges discovered accidentally by patients themselves is the most common early sign. Ninety-one percent of Chinese breast cancer patients had dense gland (95), which significantly affected the quality and effectiveness of palpation examination. For the surveillance of high-risk female carriers, in addition to regular breast self-examination and clinical breast examination, X-ray combined with ultrasound and magnetic resonance imaging (MRI) are usually selected as the methods recommended for women aged >40 years to detect early signs of breast cancer in China (96). Given that Chinese women have dense breasts and many younger patients with *BRCA1/2* mutated breast cancer, mammography screening has a lower sensitivity. A prospective study comparing different screening methods for patients with *BRCA1/2* mutations found the sensitivity of 77% with MRI compared with 36% with mammography and 33% with ultrasound (97–99).

Regular pelvic examination, tumor marker CA125 detection and transvaginal ultrasound are the methods recommended for detecting early signs of ovarian cancer (88). Annual prostate-specific antigen (PSA) testing and digital rectal examination are recommended for prostate cancer screening and surveillance, especially for *BRCA1* carriers (8). A study showed that multiparameter MRI has high diagnostic efficacy for *BRCA1* or *BRCA2* mutated prostate cancer patients. As soon as PSA elevation is detected, multiparameter MRI is recommended for *BRCA1* or *BRCA2* mutation carriers aged >55 years for further diagnosis (100). Besides, annual imaging examinations can be considered to prevent pancreatic cancer for *BRCA2* carriers, although the efficacy of this approach remains to be validated (88). The recommended starting age for monitoring breast cancer, ovarian cancer, prostate cancer and pancreatic cancer is 25, 30, 40 and 50 years, respectively, or ten years earlier than the earliest confirmed case in the family (81, 88, 94, 101).

Many studies confirmed that for *BRCA* mutation carriers, chemoprevention or surgical prevention play an important role in reducing the occurrence of HBOC (102–104). In high-risk women, prophylactic mastectomy can reduce the incidence of breast cancer by 90% and the mortality rate by 81% (103). A study showed that 23.8% and 32% of patients chose prophylactic mastectomy and prophylactic salpingo-oophorectomy; more than 17% of healthy carriers also had prophylactic surgery in Hongkong, China (102). In mainland China, however, healthy carriers and surgeons are more cautious about choosing prophylactic surgery. Only one study reported that three healthy carriers with deleterious *BRCA1/2* variant underwent prophylactic nipple-sparing mastectomy (105). Breast cancer patients carrying *BRCA1/2* deleterious variants had a 4.52-fold and 5.54-fold increased risk of contra-lateral breast cancer, respectively, compared to non-carriers (106). Preventive contra-lateral prophylactic mastectomy can be an optimal selection for *BRCA1/2* mutated breast cancer patients in China (9). Risk-reducing salpingo-oophorectomy, which can significantly reduce the risk of breast, ovarian, and fallopian tube cancers, is recommended for high-risk women after childbirth to prevent ovarian cancer (7, 107).

Studies found that *BRCA1/2*-mutated patients are more likely to benefit from platinum-based chemotherapy (108–110). Since DNA damage caused by platinum-based drugs requires DNA homologous recombination for repair, the functional defects caused by mutations in the *BRCA1/2* gene make tumor cells more sensitive to platinum-based drugs. The TNT phase III trial compared the efficacy between carboplatin and docetaxel in unselected advanced TNBC. In the germline *BRCA1/2*-mutated subgroup, the objective response rate with carboplatin was 2-fold higher than it with docetaxel (68% *vs*. 33%) (110). Recently, cancer patients with *BRCA* mutation could be benefited from poly (ADP-ribose) polymerase (PARP) targeted therapy due to the increased sensitivity to PARP inhibitors (62, 111). PARP inhibitor specifically causes the death of cancer cells with *BRCA1/2* mutations through the “synthetic lethal effect” (112). The OlympiA trial has confirmed the efficacy of PARP inhibitors in the adjuvant treatment of early-stage *BRCA1/2*-mutated breast cancer (113), while the OlympiAD trial, as well as many other phase III clinical trials, have proved the role of PARP inhibitors in advanced *BRCA1/2*-mutated breast cancer (114, 115). PARP inhibitors are widely used for *BRCA1/2*-mutated ovarian cancer patients as maintenance therapy in China based on the results of several phase III trials, including SOLO-1, SOLO-2, PAOLA-1, PRIMA and NOVA (116–120). PARP inhibitor olaparib is recommended for metastatic castration-resistant prostate cancer patients based on the PROfound trial. The phase III PROfound study showed a more prolonged imaging-based progression-free survival in the olaparib group compared with the control group (median, 7.4 months *vs*. 3.6 months) (121). PARP inhibitors are increasingly used to treat *BRCA*-mutated patients, but whether they can be used for prevention needs further investigation.

Chemoprevention for cancer-free *BRCA1/2* carriers remains controversial. Only a small retrospective study has shown that tamoxifen, a selective estrogen receptor modulator, reduces the risk of breast cancer in healthy carriers of *BRCA2* mutations by 62%. But it is unclear whether it has a preventive effect in *BRCA1*-mutated healthy carriers (122). The evidence is not enough to support tamoxifen as a prevention strategy for healthy *BRCA1/2* mutated carriers (9). Oral contraceptives have proven preventive efficacy for ovarian cancer with a family history. However, it is controversial whether oral contraceptives increase the risk of breast cancer in *BRCA1/2* mutation carriers (123).

## Conclusion and future perspective

Taken together, germline *BRCA1/2* mutations are common in Chinese patients with hereditary breast, ovarian, prostate and pancreatic cancers. Because of its ethnic specificity, the unique features in the spectrum of *BRCA* mutations have already been revealed but the extension of the sequencing efforts to the whole Chinese population remains yet to be achieved. Many Chinese consensuses today recommend *BRCA1/2* genetic testing for cancer patients only. Regarding the prevalence in healthy populations, approximately one in every 300 healthy Chinese is a *BRCA1/2* mutation carrier (12, 15). *BRCA* mutation-related cancer is one of the most preventable cancers. Whether or not to perform population screening should not solely be based on cost-effectiveness but should also consider more non-cost factors such as social, political, public interest and patients’ benefits. Under the current political and economic conditions in China, to achieve early prevention of *BRCA* mutation carriers, we recommend that the criteria be relaxed and all Chinese diagnosed with breast, ovarian, pancreatic or prostate cancer, as well as healthy individuals with a clear family history, should undergo *BRCA1/2* genetic testing to provide a risk assessment. Subsequently, preventive measures such as regular surveillance, chemoprevention or surgical prevention for mutation carriers are recommended after authentic genetic counseling.

Evidence had shown that relying on personal and family history may not be sufficient to determine the risk for *BRCA1/2* variants (20). Population *BRCA* screening is considered the trend in the near future (124, 125). Thus, a growing number of healthy individuals harboring pathogenic mutations can be identified for cancer prevention. Population screening for carriers with *BRCA* germline mutations in the Chinese population is highly warranted to promote prevention, early detection, early diagnosis, and timely treatment of *BRCA* mutation-related cancers, which may increase 5-year survival for *BRCA* mutation-related cancer patients. Also, the ethical, psychological and legal issues cannot be ignored.

## Author contributions

TC were responsible for the study concept and design. HL, MZ, LZ, KH, X-jW and TC drafted the manuscript, and all authors revised it for important intellectual content. The work reported in the paper has been performed by the authors, unless clearly specified in the text. All authors contributed to the article and approved the submitted version.

## Funding

This work was supported by grants from National Key Research-Development Program of China (2019YFE0198800), Key Research-Development Program of Zhejiang Province (2017C03013), Ten-Thousand Talents Plan of Zhejiang Province (2021R52020), and Start-up Funds for Recruited Talents in Zhejiang Cancer Hospital. The funding agencies had no role in the design and conduct of the study; collection, management, analysis, and interpretation of the data; preparation, review, or approval of the manuscript; and decision to submit the manuscript for publication.

## Conflict of interest

The authors declare that the research was conducted in the absence of any commercial or financial relationships that could be construed as a potential conflict of interest.

## Publisher’s note

All claims expressed in this article are solely those of the authors and do not necessarily represent those of their affiliated organizations, or those of the publisher, the editors and the reviewers. Any product that may be evaluated in this article, or claim that may be made by its manufacturer, is not guaranteed or endorsed by the publisher.

## Abbreviations

CFCSG-database, Chinese Familial & Hereditary Cancer Susceptibility Gene Mutation database; ESMO, the European Society of Medical Oncology; HBOC, hereditary breast and ovarian cancer syndrome; LGRs, large genomic rearrangements; MLPA, multiplex ligation-dependent probe amplification; MRI, magnetic resonance imaging; NCCN, National Comprehensive Cancer Network; NGS, next-generation sequencing; PARP, poly (ADP-ribose) polymerase.

PSA, prostate-specific antigen; SGO, the Society of Gynecologic Oncology; VUS, variants of uncertain significance; WES, whole-exome sequencing; WGS, whole-genome sequencing.
